# Hematopoietic Aging and Leukemia: Mechanistic and Therapeutic Insights

**DOI:** 10.3390/ijms27094043

**Published:** 2026-04-30

**Authors:** Zhihui Li, Hao Zhang, Nuo Cheng, Hong Li, Xiaoling Wang, Jingbo Shao

**Affiliations:** 1 Department of Hematology and Oncology, Shanghai Children’s Hospital, School of Medicine, Shanghai Jiao Tong University, Shanghai 200062, China; 2 Department of Geriatrics, Medical Center on Aging of Ruijin Hospital, School of Medicine, Shanghai Jiao Tong University, Shanghai 200025, China; 3 Cancer Research UK Cambridge Institute, University of Cambridge, Cambridge CB2 0RE, UK; 4 Department of Reproductive Medical Center, Ruijin Hospital, School of Medicine, Shanghai Jiao Tong University, Shanghai 200025, China

**Keywords:** hematopoiesis, aging, chronic inflammation, bone marrow niche, leukemia

## Abstract

Aging profoundly alters hematopoiesis by impairing stem cell self-renewal, skewing lineage differentiation, and remodeling immune and stromal compartments within the bone marrow. Consequently, these changes contribute to an increased susceptibility to leukemia. Conversely, leukemia contributes to systemic aging. Although the connection between hematopoietic aging and leukemogenesis has been well-recognized, the precise molecular and microenvironmental mechanisms underlying this association remain poorly elucidated. In recent years, emerging studies have identified altered clonal dynamics, chronic inflammation, and niche-dependent metabolic remodeling as major contributors to malignant transformation. Building on these findings, we synthesize current insights into how aging reprograms the hematopoietic ecosystem to promote leukemic initiation and progression, and furthermore, discuss potential strategies to counteract these processes by targeting aging-related pathways.

## 1. Introduction

**Figure 1:**
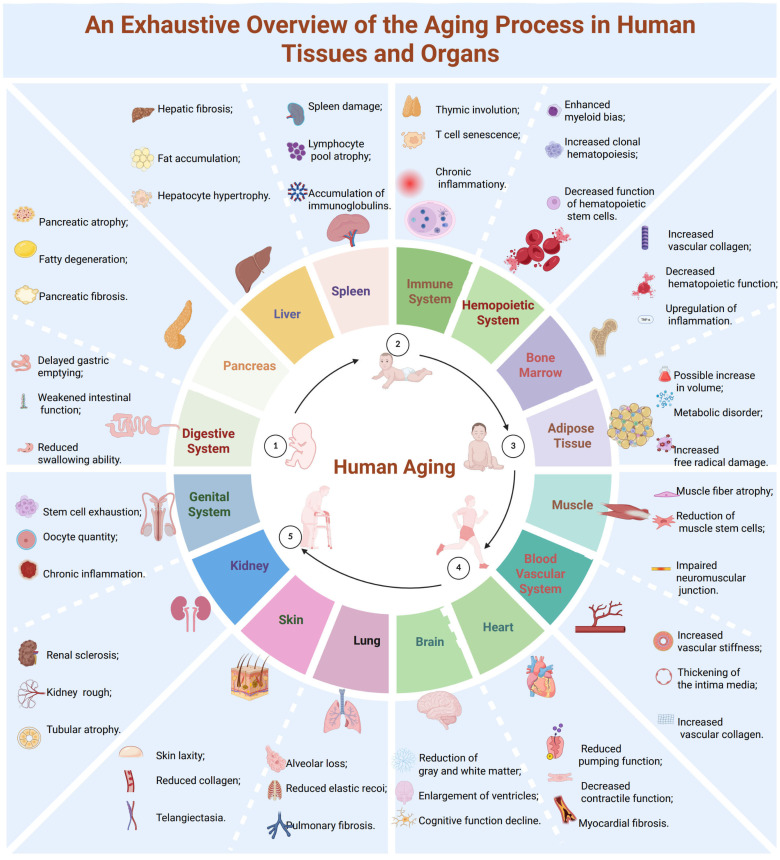
Schematic Overview of Age-Related Structural and Functional Changes in Human Organs and Tissues. Aging induces key structural and functional changes across major organ systems. These changes include cerebral atrophy and cognitive decline in the brain; reduced contractility and fibrosis in the heart; alveolar loss and decreased lung elasticity; hepatic steatosis and fibrosis; thymic involution and immune senescence; sarcopenia and neuromuscular degeneration; vascular stiffening and intimal thickening; and alterations in hematopoietic function, including clonal expansion. Collectively, these processes drive a progressive decline in physiological resilience and overall organ function with advancing age. (Created in BioRender. Zhihui Li. (2026) https://app.biorender.com/illustrations/canvas-beta/674d168c5bfa5ed49b4aea7e).

Aging is an inevitable biological process characterized by progressive decline at the molecular, cellular, tissue, and organ levels, leading to reduced physiological reserve and increased vulnerability to disease [1]. Virtually all organ systems are affected (Figure 1; Table 1), and substantial heterogeneity exists in aging trajectories across the eleven major human organ systems [2,3]. Emerging evidence suggests that preservation of youthful brain and immune system function is a key determinant of healthspan extension [4,5,6,7].

In line with this view, aging has traditionally been regarded as a passive risk factor, a growing body of evidence supports a more dynamic paradigm in which aging actively drives disease initiation and progression [4]. In the context of hematologic malignancies, this shift in perspective is particularly relevant, as the hematopoietic system serves as the foundation of the immune system and appears especially susceptible to aging-related perturbations. Because it must sustain continuous regeneration throughout life and depends on a tightly regulated bone marrow niche, even subtle molecular or cellular disruptions can accumulate and gradually undermining stem cell function and compromising immune and systemic homeostasis [8]. Hematopoietic aging involves progressive decline in stem cell function, structural and functional remodeling of the bone marrow niche, and chronic low-grade inflammation [8,9,10]. Importantly, these aging-associated alterations do not merely reflect functional decline but actively reshape the hematopoietic environment in ways that promote malignant transformation.

Consistent with this, mounting evidence now indicates that hematopoietic aging and leukemogenesis are mechanistically intertwined. Age-related shifts in signaling, stem cell competition, and clonal diversity then contribute to leukemia progression and influence clinical outcomes [11]. Importantly, leukemia itself may further accelerate both hematopoietic and systemic aging, creating a self-reinforcing pathological cycle [8,12].

Beyond disease initiation, the impact of aging extends to therapeutic response. These clinical challenges are underpinned by dynamic changes in aging-associated biomarkers during leukemia development, which both reflect and contribute to the progressive deterioration of hematopoietic and systemic function [13]. This highlights the need for aging-informed biomarkers and risk stratification frameworks to guide personalized treatment strategies. Accordingly, hematopoietic aging represents both a biological precursor and a clinical modifier of leukemia. Emerging interventions-ranging from senolytics, metabolic modulation, to epigenetic therapies-combined with multi-omics and systems biology approaches, hold promise for mitigating age-related vulnerabilities [14,15]. Despite growing recognition of the link between hematopoietic aging and leukemogenesis, the underlying molecular and microenvironmental mechanisms remain insufficiently resolved. In this review, we propose that hematopoietic aging should be viewed not merely as a risk factor but as a therapeutically actionable driver of leukemogenesis, and we identify key vulnerabilities that may inform targeted strategies.

## 2. Biological Features of Hematopoietic Aging

Aging impairs hematopoietic function and homeostasis, reducing blood and immune output and increasing susceptibility to anemia, immunodeficiency, and hematologic malignancies. Consequently, age-related changes in HSCs, the marrow niche, and immune regulation have become central research themes.

### 2.1. Aging-Associated Changes in Hematopoietic Stem Cells

HSCs sustain lifelong blood production, but aging alters their number, differentiation potential, and function. Some HSC clones expand in vivo, a phenomenon known as clonal hematopoiesis (CH) [16]. When such clones harbor specific mutations without overt disease, they are classified as clonal hematopoiesis of indeterminate potential (CHIP) [17].

Importantly, CHIP should be distinguished from clonal cytopenia of undetermined significance (CCUS), in which similar somatic mutations are present but are accompanied by persistent cytopenias. Compared with CHIP, CCUS carries a substantially higher risk of progression to myelodysplastic syndromes (MDSs) and acute myeloid leukemia (AML), underscoring the clinical heterogeneity of clonal hematopoiesis. Conceptually, CHIP and CCUS lie along a continuum of clonal evolution, with CCUS reflecting the transition from molecular alterations to overt hematopoietic dysfunction, thereby enabling improved risk stratification [18]. CHIP is typically characterized by driver mutations with a variant allele frequency (VAF)-the proportion of sequencing reads supporting a mutation. Large cohort and longitudinal studies show that CHIP prevalence increases markedly with age; for example, CHIP is detected in approximately 10–15% of individuals older than 70 years in population-based sequencing studies. However, exact estimates vary depending on sequencing methods and VAF thresholds. More sensitive sequencing approaches generally detect CHIP in a larger proportion of individuals, highlighting the influence of methodological sensitivity on reported prevalence [19,20,21]. Recent studies further indicate that clonal hematopoiesis is a dynamic process rather than a static state, with the relative contribution of high-risk mutant clones evolving over time [16].

Functionally, aged bone marrow often shows increased HSC numbers, but a decline in regenerative capacity. Functional studies consistently reveal reduced self-renewal, long-term regenerative capacity, and a pronounced myeloid bias. This skewing limits lymphoid output and impairs adaptive immunity, indicating that functional decline outweighs numerical expansion [13,22,23].

At the mechanistic level, these changes are driven by the lifelong accumulation of stochastic somatic mutations in HSCs, coupled with impaired DNA repair pathways, which together provide the substrate for clonal selection and CHIP emergence [20,24]. Impaired DNA repair leads to progressive DNA damage accumulation (e.g., γH2AX and 53BP1 foci). Telomere attrition and mitochondrial dysfunction, including increased reactive oxygen species (ROS), further exacerbate genomic instability and HSC dysfunction [25,26,27,28]. In turn, elevated ROS and persistent DNA damage disrupt epigenetic regulation and gene regulatory networks. This reduces transcriptional fidelity and adaptive capacity, contributing to age-associated loss of regulatory information [29,30,31]. These alterations confer selective advantages to clones harboring mutations in genes such as DNMT3A, TET2, ASXL1, and splicing factors (e.g., SF3B1, SRSF2, U2AF1), driving their expansion. Mechanistically, DNMT3A mutations promote HSC self-renewal through epigenetic dysregulation, whereas TET2 loss enhances inflammatory signaling and confers a competitive advantage within the aged niche; both may also impair DNA damage responses, facilitating additional mutation accumulation. Emerging evidence suggests that targeting key regulatory nodes (e.g., SIRT1, NRF2, TP53, AMPK, and FOXO3) may partially restore network integrity and regulatory function, offering potential avenues for rejuvenation strategies [32,33,34].

Collectively, these interconnected processes drive clonal expansion and lineage skewing by disrupting regulatory networks, ultimately leading to age-associated hematopoietic dysfunction and increased susceptibility to hematologic malignancies.

### 2.2. Age-Related Remodeling of the Hematopoietic Niche

HSC function is tightly regulated by the bone marrow niche, which comprises mesenchymal stem cells (MSCs), endothelial cells, osteoblasts, adipocytes, immune cells, nerve fibers, cytokines, and extracellular matrix (ECM) components. Aging selectively remodels this microenvironment: MSCs support and osteogenesis decline, whereas adipogenesis and marrow adiposity increase, leading to reduced support for lymphoid-biased HSCs and altered niche metabolic outputs. Immune cells, including macrophages, T cells, and natural killer cells, exhibit age-related functional decline, contributing to chronic low-grade inflammation and impaired antitumor surveillance. Bone marrow sympathetic nerve fibers also degenerate with age, disrupting HSC trafficking and circadian regulation [27,35].

Endothelial cells also lose barrier integrity and angiogenic capacity, resulting in reduced microvascular density. This, in turn, impairs HSC localization by decreasing homing and retention factors such as CXCL12 and VCAM1, ultimately diminishing HSC quiescence and regenerative capacity [36,37,38,39]. Recent spatial transcriptomic and proteomic imaging studies show that age-associated niche inflammation is regionally heterogeneous. Periarteriolar niches exhibit elevated NF-κB and inflammasome signaling compared with perisinusoidal regions, promoting lymphoid progenitor depletion, whereas myeloid-supportive niches remain relatively preserved. These localized alterations coincide with systemic cytokine shifts, including increased IL-6 and TNF-α and decreased SCF and CXCL12, linking niche inflammation, immune cell dysfunction, and impaired HSC output [40,41,42].

Concomitantly, the ECM component of the marrow niche exhibits age-related remodeling. Progressive stiffening of the stromal scaffold perturbs integrin-mediated adhesion and mechanical signal transduction pathways, biasing HSC differentiation toward the myeloid lineage and reducing self-renewal capacity. Experimental increases in matrix stiffness recapitulate these lineage biases [43,44].

Importantly, heterochronic transplantation demonstrates the rejuvenating power of a young hematopoietic niche. Exposure of aged HSCs to youthful stromal or systemic environments restores CXCL12 expression, engraftment efficiency, and quiescence, highlighting the critical role of extrinsic niche cues, including stromal, immune, and neural components, in hematopoietic aging [45].

Aging-associated alterations in hematopoiesis represent a coordinated causal network rather than isolated events. Elevated ROS initiate DNA damage and genomic instability, driving epigenetic dysregulation and senescence, which promote SASP and chronic inflammation. This inflammatory state remodels the bone marrow niche and impairs HSC function, while the dysfunctional niche further amplifies oxidative and metabolic stress, forming a self-reinforcing loop that accelerates hematopoietic aging and leukemogenesis.

**Figure 2:**
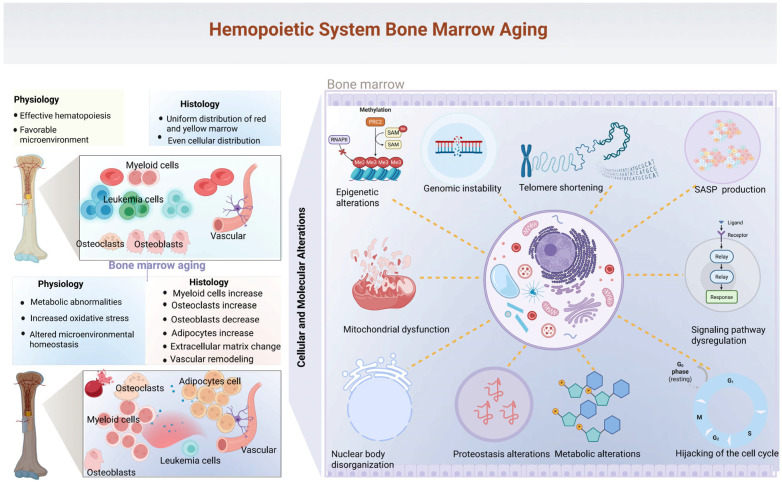
Cellular and molecular mechanisms of bone marrow aging. Aged bone marrow exhibits key structural, functional, and molecular alterations. Compared with healthy marrow, aging is characterized by skewed myeloid expansion, disrupted osteoblast–osteoclast balance, widespread cellular dysfunction and so on. Underlying mechanisms include epigenetic modifications, genomic instability, telomere attrition, mitochondrial dysfunction, metabolic reprogramming, and SASP production, which collectively compromise hematopoietic homeostasis and the supportive bone marrow microenvironment. (Created in BioRender. Zhihui Li. (2026) https://app.biorender.com/illustrations/canvas-beta/675687823d433c3ec33c9038).

Within this cascade, epigenetic drift, telomere attrition, mitochondrial reprogramming, proteostasis collapse, and signaling dysregulation act in concert with niche remodeling, immune dysfunction, and neural deterioration, collectively exhausting hematopoietic stem and progenitor cells and driving systemic immune dysregulation (Figure 2).

### 2.3. Aging-Driven Immune Remodeling

Hematopoietic aging markedly reshapes immune composition and function, a phenomenon often called immunosenescence. Evidence from large-scale human and animal studies shows that aging skews differentiation toward myeloid lineages, increasing neutrophil and monocyte numbers while simultaneously impairing their effector functions [46]. For example, neutrophils from older adults exhibit reduced chemotaxis, diminished phagocytosis and lower microbicidal activity, even as their absolute counts remain stable or rise [47,48]. Natural killer (NK) cells, though often increased in number in the elderly, show reduced cytotoxicity, decreased cytokine secretion and upregulation of inhibitory markers, undermining immune surveillance of senescent or malignant cells [49,50,51]. On the adaptive-immune side, B-cell lymphopoiesis declines with age: fewer naïve B cells are produced, antibody diversity contracts, and the frequency of late-stage exhausted memory B cells rises [52,53]. Thymic involution further diminishes output of naïve T cells, shifting peripheral T-cell populations toward memory phenotypes with increased expression of inhibitory receptors such as PD-1 and reduced T-cell receptor repertoire diversity [54,55].

In addition to these cellular alterations, aging is associated with a systemic pro-inflammatory state characterized by persistently elevated cytokines, including IL-1, IL-6, CRP, and TNF-α, which functions not merely as a correlated feature but as an upstream driver of hematopoietic dysfunction. Chronic exposure to these cytokines reshapes the bone marrow niche and directly acts on HSCs, leading to skewed lineage differentiation, reduced self-renewal capacity, and progressive functional exhaustion. While this work highlights IL-1 signaling as a key mediator of HSC aging, its role extends mechanistically to reinforcing inflammatory feedback loops, modulating epigenetic states, and disrupting niche support, thereby linking systemic inflammation to intrinsic stem cell decline. However, these mechanisms may not be universally applicable, as the study does not fully address conflicting findings across different leukemia contexts or account for the complex interplay between systemic and niche-specific effects [56]. Importantly, these processes are not independent but form an interconnected, feed-forward cycle in which chronic inflammation amplifies HSC dysfunction, which in turn further perturbs immune regulation. Collectively, these changes reflect a decline in immune competence and resilience, leading to impaired host defense and altered hematopoietic–immune system interactions in older individuals.

### 2.4. Peripheral Blood Lineage Aging

Aging impairs function and structure across all peripheral blood lineages, not just immune cells. In erythrocytes, it appears as cumulative oxidative and metabolic stress. Mitochondrial dysfunction and reduced glycolysis raise ROS levels, driving lipid peroxidation and accumulation of markers like MDA and PLOOH. These insults weaken membrane lipids and the cytoskeleton, reducing deformability and making aged red cells more prone to clearance. At the same time, “don’t-eat-me” signals such as CD47, CD44, and CD147 are diminished or altered, while band-3 clustering further promotes pro-phagocytic signaling [57,58]. Together, these changes shorten erythrocyte lifespan, impair oxygen transport, and likely contribute to the high prevalence of unexplained anemia in older adults. Age-related shifts in red-cell morphology, biochemistry, and production have been consistently observed [59].

Leukocyte aging is driven by cumulative genotoxic and metabolic stress. DNA damage and replication stress activate the ATM/ATR–CHK1/CHK2 axis, inducing p16^INK4a–p21^CIP1–mediated cell-cycle arrest and triggering a pro-inflammatory SASP [60]. In neutrophils, ROS-induced DNA damage activates ATM, modulating cytokine production and apoptosis, linking oxidative bursts to age-related innate immune decline. Elevated mitochondrial ROS and dysfunction further amplify DNA damage signaling and genomic instability [61,62]. Aged macrophages show defective mitochondrial turnover, increased mtDNA leakage, and higher ROS, correlating with impaired phagocytosis and exaggerated inflammation [63,64]. Memory CD4+ T cells from older donors accumulate mitochondria and ROS [65], promoting secretion of IFN-γ, IL-6, and IL-8, connecting mitochondrial dysregulation to immune remodeling [66]. Concurrently, telomere attrition, impaired DNA repair, and γH2AX foci in T cells associate with upregulation of p53-dependent cell cycle inhibitors and reduced proliferation, highlighting the convergence of DNA damage and senescence pathways [67]. These changes diminish leukocyte proliferative reserve, alter morphology and cytokine output, and impair immune surveillance, paralleling the clinical features of immunosenescence.

**Figure 3:**
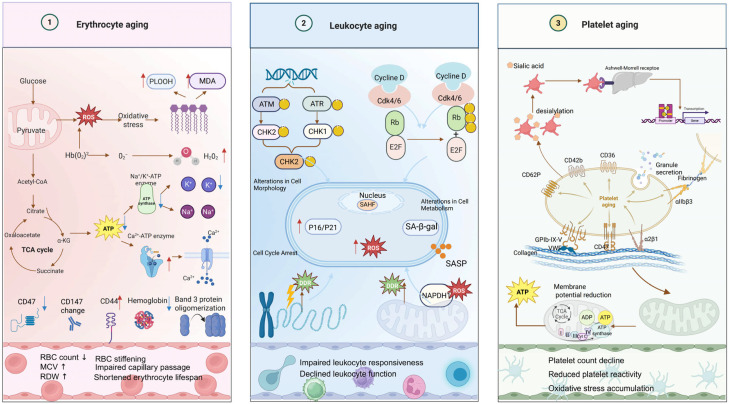
Molecular and metabolic mechanisms of blood cell aging. Key pathways driving the aging of erythrocytes, leukocytes, and platelets are illustrated. Erythrocytes show oxidative hemoglobin damage, membrane alterations, metabolic shifts, and senescence signals (e.g., CD47). Leukocytes exhibit DNA damage response, cell cycle inhibitor activation (p16/p21), and senescence-associated features including SA-β-gal and SASP. Platelets display altered receptor expression, granule secretion, desialylation, and increased adhesion. Shared mechanisms across all cell types include mitochondrial dysfunction, ROS accumulation, and metabolic reprogramming. (Red arrows represent an increase, whereas blue arrows represent a decrease). (Created in BioRender Zhihui Li. (2026) https://app.biorender.com/illustrations/canvas-beta/67da804af03208272e347067).

Platelet aging reflects both intrinsic mitochondrial decline and extrinsic clearance. Desialylation of surface glycoproteins (CD62P, CD42b, CD36, GPIb-IX-V) enhances recognition by the hepatic Ashwell–Morrell receptor, accelerating platelet removal. Mitochondrial dysfunction—marked by loss of membrane potential, ATP depletion, and elevated ROS—impairs hemostatic control and promotes aberrant activation, increasing aggregation and thrombosis risk (Figure 3). These interconnected changes help explain the higher incidence of thromboembolic events in older individuals, linking hematopoietic aging to cardiovascular disease [68,69,70,71,72,73].

Collectively, such lineage-specific alterations form a hierarchical cascade—from stem cells to circulating elements—reducing immune defense, oxygen delivery, and hemostatic stability, while fostering systemic inflammation and susceptibility to malignancy.

## 3. Aging-Associated Alterations in Leukemia Initiation and Clinical Features

**Figure 4:**
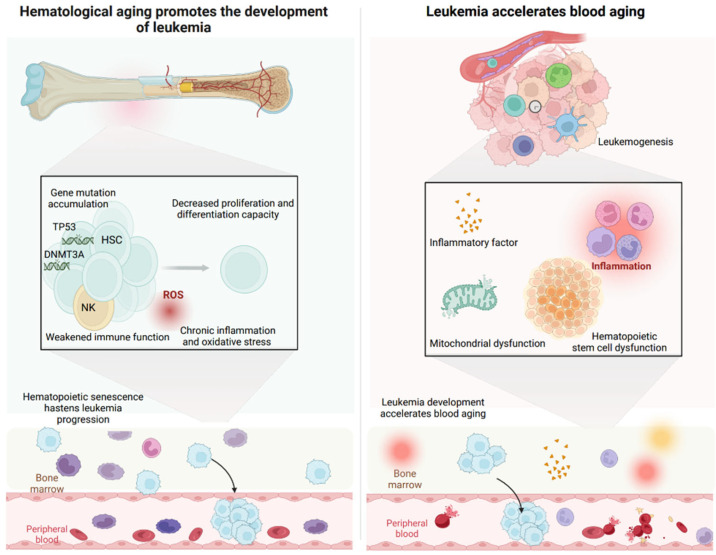
Interplay between hematopoietic aging and leukemia development. The bidirectional relationship between hematopoietic aging and leukemogenesis is illustrated. Aging promotes leukemia via gene mutations (e.g., TP53, DNMT3A), chronic inflammation, oxidative stress (ROS), and impaired hematopoietic proliferation and differentiation. Conversely, leukemia accelerates blood and immune aging by inducing mitochondrial dysfunction, releasing inflammatory factors, and disrupting normal hematopoiesis in bone marrow and peripheral blood. This vicious cycle may promote leukemia progression and exacerbate age-related decline in hematopoietic and immune function. The arrows indicate the migration of leukemic cells from the bone marrow into the peripheral blood. (Created in BioRender. Zhihui Li. (2026) https://app.biorender.com/illustrations/canvas-beta/67b594f2670a6a23ca9f2386).

Aging profoundly reshapes hematopoiesis and directly influences leukemia initiation and clinical behavior. Accumulating driver mutations, chronic inflammation, oxidative stress, and mitochondrial dysfunction exhaust HSCs and shift them toward a myeloid-biased, immunosuppressed state that heightens leukemic susceptibility (Table 2). These aging-associated alterations not only facilitate malignant transformation but also shape the clinical features of leukemia including higher disease incidence, increased genomic instability, and poorer therapeutic responses. Once established, leukemia further accelerates hematopoietic aging through genomic instability and inflammatory feedback (Figure 4).

### 3.1. Aging-Induced Myeloid Bias in Accelerating Leukemia Development

Aging-induced myeloid bias marks a fundamental shift in HSC behavior that fuels leukemia development [74,75]. Over time, HSCs accumulate somatic mutations, suffer mitochondrial decline, and experience chronic inflammatory stress, collectively weakening lymphoid output while favoring myeloid-dominant differentiation. This skew not only dampens immune surveillance but also enlarges the pool of myeloid progenitors prone to malignant transformation. Mutations in genes such as ASXL1, RUNX1, SF3B1, SRSF2, and CBL reinforce this biased state, promoting myeloid commitment and creating fertile ground for clonal expansion [74,76,77].

Notably, while the decline in lymphoid output and expansion of myeloid lineages are well established, the impact of aging on erythroid differentiation remains more controversial. Some studies report an expansion of erythroid progenitors in aged bone marrow, potentially reflecting compensatory responses to anemia or stress erythropoiesis, whereas others demonstrate reduced erythroid output and impaired erythropoietic capacity due to stem cell dysfunction and niche alterations. These discrepancies likely arise from differences in experimental models, physiological context, and disease states, underscoring the complexity and heterogeneity of lineage commitment during hematopoietic aging [78].

While less pronounced in children, mild myeloid skewing can arise from congenital mutations or chromosomal abnormalities, subtly predisposing to myeloid leukemia. The aged bone marrow niche—shaped by senescent stromal cells, oxidative stress, and altered cytokine signaling—further amplifies this bias, giving preleukemic clones a selective edge [27]. Once leukemia emerges, it feeds back to accelerate hematopoietic aging through genomic instability, inflammation, and niche deterioration, forming a self-reinforcing cycle in which myeloid bias contributes to leukemia, and leukemia accelerates aging [79].

### 3.2. DNA Damage and Epigenetic Reprogramming in Leukemia Initiation

Aging HSCs accumulate DNA damage from telomere attrition, replication stress, and reactive oxygen species, while DNA repair capacity declines [80]. Cells surviving with incomplete repair may acquire oncogenic mutations, providing an initial “hit” toward leukemia [81]. Concurrently, epigenetic drift—including altered DNA methylation, histone modifications, and chromatin accessibility—reshapes gene expression, conferring a selective advantage to mutant clones. Mutations in DNMT3A, TET2, and ASXL1, common to both clonal hematopoiesis and leukemia, link age-associated hematopoietic changes to malignant transformation [82].

Recent studies reveal crosstalk between DNA damage and epigenetic reprogramming: TET2 mutations impair double-strand break repair, favoring error-prone pathways, whereas DNMT3A mutations bias repair toward homologous recombination, influencing leukemic risk [83]. Additional layers—RNA m^6^A modifications, metabolic reprogramming, and 3D chromatin dynamics—further destabilize genomic integrity and alter HSC fate, skewing clonal expansion [84]. Together, the convergence of DNA damage, declining repair fidelity, epigenetic and epitranscriptomic remodeling, and altered differentiation creates a permissive environment for benign clones to evolve into overt leukemia.

### 3.3. Metabolic Reprogramming in Leukemia Stem Cell Survival

As the hematopoietic system ages, the metabolic programming of HSC undergoes profound rewiring. Recent work in 2025 has generated the first comprehensive “map” of human HSC metabolism, revealing that human HSCs are significantly less metabolically active than their differentiated progeny, relying predominantly on glycolysis, maintaining relatively quiescent mitochondria, and utilizing fewer metabolites for energy production and amino acid synthesis. This study provides detailed metabolic profiling of human HSCs across aging and leukemia, but it is limited by small sample sizes, potential heterogeneity in AML patient samples, and a lack of functional validation to confirm whether the observed metabolic changes causally influence stem cell function or leukemogenesis [85]. Under youthful conditions, HSCs typically rely on glycolysis and maintain relatively quiescent mitochondria, limiting oxidative phosphorylation (OXPHOS) and ROS production. With advancing age, however, a shift occurs: HSCs increasingly engage OXPHOS, mitochondrial activity rises, and ROS accumulate alongside mitochondrial dysfunction. Mechanistically, elevated ROS activate DNA damage responses and stress signaling pathways (e.g., p38 MAPK, ATM/ATR), which contribute to stem cell attrition and favor the emergence of competitive clones [29,86].

In this aged metabolic milieu, leukemia stem cells (LSCs) exploit the altered landscape for survival and growth. LSCs are adept at harnessing enhanced fatty acid oxidation (FAO) and upregulating key enzymes such as CPT1A to maintain ATP production under metabolic stress, thereby sustaining self-renewal and chemoresistance [87,88]. Recent reviews of hematologic malignancies emphasize that LSCs’ reliance on OXPHOS and mitochondrial function is integral to their maintenance and resistance to therapy. Recent studies further highlight that loss of TET2 function specifically reshapes HSC metabolism, with TET2-deficient cells exhibiting upregulation of genes involved in glycolysis and OXPHOS, expansion of the mitochondrial network, and enhanced oxidative metabolism compared with wild-type counterparts. Mechanistic interrogation using in vitro metabolic flux assays and in vivo transplantation models indicates that these metabolic alterations confer competitive advantages via enhanced redox buffering, NADPH production, and ATP generation, which together promote clonal dominance and leukemogenic potential. Such rewiring not only promotes clonal dominance but also identifies potential metabolic vulnerabilities: targeting glycolytic flux, mitochondrial oxidative metabolism, or pathways sustaining redox homeostasis may constrain the expansion of high-risk clones before overt leukemic transformation. Together, these findings underscore that metabolic reprogramming—both age-associated and mutation-driven—represents a central driver of HSC fitness, clonal selection, and leukemia progression, and offers actionable targets for therapeutic intervention in older adults [89].

### 3.4. Aged Bone Marrow Niche in Leukemia Development and Progression

The bone marrow niche plays a central role in regulating leukemogenesis, and aging profoundly alters its composition and function. Chronic low-grade inflammation, or “inflammaging,” leads to elevated levels of cytokines such as IL-6, TNF-α, and IL-1β, which support the expansion of leukemic clones while reducing the competitive fitness of normal HSCs [88,90]. Structural and vascular changes in the aging marrow—including increased adiposity, reduced osteogenesis, and diminished vascular density—further weaken support for normal hematopoiesis and create conditions favorable for LSC survival [91].

Aging also reshapes the immune microenvironment. Macrophages in older marrow acquire a pro-inflammatory, dysfunctional state, T-cell and NK cell activity declines, and populations of regulatory T cells and myeloid-derived suppressor cells increase [92,93]. Together, these changes impair immune surveillance, allowing malignant clones to evade clearance. At the same time, stromal cells in the niche undergo metabolic and epigenetic reprogramming: mesenchymal stromal cells favor adipogenic over osteogenic differentiation, reduce secretion of supportive factors such as CXCL12, and increase inflammatory chemokines, enhancing LSC homing, retention, and survival [94]. Altered interactions between LSCs and adipocyte-rich regions further promote a myeloid-biased hematopoiesis, reinforcing leukemia progression [95].

These combined alterations—chronic inflammation, immune decline, stromal remodeling, and metabolic changes—create a permissive environment that both supports LSC survival and contributes to therapeutic resistance. The aging bone marrow niche thus shifts from a supportive environment for normal hematopoiesis to a landscape that actively may facilitate leukemia initiation and progression, highlighting the importance of targeting niche-derived signals in strategies to prevent or treat age-associated leukemia.

### 3.5. Age-Dependent Clinical Features and Prognosis of Leukemia

Aging not only shapes leukemia mechanistically but also dictates clinical presentation and outcome. In Acute Myeloid Leukemia (AML), younger patients (typically < 60 years) more often present with favorable genetic forms such as core-binding-factor AML (CBF-AML: t(8;21), inv(16)) or acute promyelocytic leukemia (APL; PML::RARA) [96]. These cases are characterized by lower mutational burden, simpler karyotypes and more stable HSC clones, resulting in high chemotherapy responsiveness and relatively favorable long-term survival [97]. In contrast, older patients (≥60 years) more frequently develop high-risk AML with complex karyotypes, frequent TP53 mutations or secondary AML arising from prior myelodysplastic syndrome (MDS) or marrow injury [98].

Recent large-scale analyses reinforce this age gradient: a study of over 2800 adult AML patients showed near-linear decline in survival with increasing age—even within favorable, intermediate and adverse genetic-risk groups. Moreover, a prognostic model in intensively treated patients older than 60 identified a high-risk subgroup with only ~4% four-year survival, underscoring the biological and clinical heterogeneity driven by age [99].

Sex differences further modulate AML outcomes: men account for 55–60% of AML cases and exhibit poorer prognosis, with a 5-year overall survival (OS) of 48.8%, compared to 60.4% in women. These disparities are partly attributable to sex-specific mutational landscapes and pharmacokinetic variations, and evidence suggests that sex and aging interact to influence leukemia incidence, progression, and therapeutic response [100].

Aging influences leukemia across multiple dimensions—molecular, cellular, microenvironmental, and clinical. It drives the expansion and malignant transformation of pre-leukemic clones, shapes mutational landscapes and metabolic adaptations, remodels the bone marrow niche, and alters stem cell function and immune competence, collectively determining disease trajectory and treatment tolerance. Linking these biological changes to clinical outcomes highlights the need to translate mechanistic insights into age-informed therapeutic strategies. Considering both age and sex provides a comprehensive framework for precision therapies in elderly AML patients, targeting inflammatory signaling, metabolic reprogramming, and epigenetic dysregulation.

## 4. Impact of Aging on Leukemia Treatment Tolerability, Efficacy, and Prognosis

Hematopoietic aging drives reduced treatment tolerance and poorer responses in older patients. Linking these biological changes to clinical outcomes provides a framework for tailoring therapies and improving management in the elderly.

### 4.1. Age-Dependent Response to Conventional Therapy

Intensive chemotherapy has long been the backbone of AML therapy, yet older patients face disproportionately high risks of severe myelosuppression, infections, bleeding, and multi-organ toxicity [101,102]. These vulnerabilities stem from reduced bone marrow reserve, declining hepatic and renal function, and frequent comorbidities, often rendering standard regimens intolerable and increasing treatment-related mortality. To address this, low-intensity approaches—such as hypomethylating agents, low-dose cytarabine, and attenuated combination regimens—have been developed. While more tolerable, survival gains remain modest compared with younger patients [103].

Recent advances have shifted the landscape for older or medically unfit AML patients. Venetoclax combined with hypomethylating agents, such as azacitidine, has emerged as a frontline non-intensive standard, achieving higher complete remission rates and improved median overall survival (~14.7 vs. ~9.6 months) compared with azacitidine alone, with particular benefit in molecularly defined subgroups, including IDH1/2-mutated AML [104]. Venetoclax plus low-dose cytarabine also outperforms LDAC alone (VIALE-C) [105]. For fit older patients with secondary or therapy-related AML, CPX-351 (liposomal daunorubicin-cytarabine) demonstrates superior remission and survival versus conventional 7 + 3 regimens [106,107].

Immunotherapies and novel targeted agents are actively being investigated. Early-phase studies of anti-CD47 antibodies (e.g., magrolimab with azacitidine) show promise, especially in TP53-mutant AML, though recent phase III results have been mixed, underscoring the need for further validation [108].

Given the narrow margin between efficacy and toxicity in older adults, comprehensive geriatric assessment (CGA) and geriatric-oncology tools are increasingly used to guide treatment. Evaluating frailty, cognition, comorbidities, nutrition, and functional reserve helps tailor therapy—ranging from intensive chemotherapy to venetoclax-based regimens, CPX-351, or supportive/experimental approaches—optimizing both safety and outcomes.

In summary, although outcomes in older AML patients remain poorer than in younger cohorts, venetoclax-based non-intensive regimens improve survival, CPX-351 benefits select high-risk patients, and emerging targeted or immunotherapies expand options. Routine geriatric assessment guides treatment intensity, optimizing efficacy and safety.

### 4.2. Aging-Associated Effects on Targeted Therapy

Reduced-intensity regimens have improved treatment feasibility for older adults, yet the therapeutic landscape has been further transformed by the introduction of molecularly targeted agents. Aging, however, significantly reshapes both the genomic landscape of AML and MDS and the bone marrow microenvironment, profoundly influencing responsiveness to these precision therapies [109].

Genomic studies indicate that older patients harbor distinct mutational profiles that critically impact treatment outcomes [110]. In addition to canonical age-related lesions, TP53 mutations and alterations in spliceosome genes (SRSF2, SF3B1, U2AF1) are particularly prevalent and confer adverse biology, often driving resistance to both chemotherapy and targeted therapies [111,112]. Clinical trials, such as AGILE, have shown that genotype-guided approaches—illustrated by ivosidenib combined with azacitidine for IDH1-mutant AML—can yield meaningful survival benefits even in frail patients [113]. Likewise, FLT3 inhibitors paired with low-intensity regimens are demonstrating emerging feasibility in elderly cohorts [114].

Despite these advances, durable responses are frequently limited by adaptive resistance and protective effects of the microenvironment. Metabolic reprogramming and compensatory upregulation of MCL-1 and BCL-XL can blunt the efficacy of BCL-2 inhibition, while senescent and inflammatory alterations in the aged bone marrow niche support the persistence of resistant subclones [115]. Current research therefore emphasizes rational combination strategies, integrating molecularly targeted therapies with approaches directed at metabolism or the microenvironment. These strategies aim to surmount age-associated barriers and achieve sustained remission in older AML and MDS patients, though ongoing molecular profiling and comprehensive geriatric assessment remain essential to guide individualized treatment planning.

### 4.3. Immunosenescence and Limitations of Immunotherapy

Age-related changes in both the genome and the bone marrow microenvironment influence responses to chemotherapy and targeted therapies, but alterations in immune function add an additional layer of complexity that constrains the efficacy of emerging immunotherapies in older leukemia patients.

Immune aging limits the effectiveness of these treatments through multiple mechanisms [116]. T-cell senescence reduces the pool of naïve cells, impairs effector function, and increases inhibitory receptor expression, including PD-1 and TIM-3, thereby diminishing the impact of checkpoint blockade [117,118]. Natural killer (NK) cell cytotoxicity and CAR T-cell proliferation are similarly impaired. At the systemic level, chronic low-grade inflammation (“inflammaging”) and reduced organ reserve elevate the risk of severe adverse events, such as cytokine release syndrome, further restricting the applicability of immune-based therapies in the elderly [118].

Recent evidence offers a more nuanced perspective. A multicenter observational study found that older adults receiving immune checkpoint inhibitors (ICIs) achieved outcomes comparable to younger patients, challenging the notion that advanced age alone precludes benefit [119]. Preclinical studies indicate that increased expression of exhaustion markers on CD8^+^ T cells and impaired dendritic cell migration in aged hosts reduce ICI efficacy. Additionally, aging is associated with accumulation of senescent immune cells, expansion of immunosuppressive myeloid populations, and elevated levels of SASP cytokines, all of which foster a tumor-promoting microenvironment and may blunt the activity of CAR T or NK cell therapies [116,120].

These findings underscore that immunosenescence extends beyond a simple reduction in immune vigor. Instead, it reshapes immune composition, functional capacity, and microenvironmental interactions, highlighting the need for immunotherapy strategies that are specifically tailored to the aged immune system.

### 4.4. Biomarkers of Aging for Precision Therapy

While immunosenescence constrains the efficacy of immune-based therapies in patients, age-associated molecular, epigenetic, inflammatory, and metabolic alterations mechanistically modulate cellular signaling, DNA repair capacity, and immune effector functions, thereby shaping responses to conventional, targeted, and immunotherapeutic interventions. Blood-derived aging biomarkers are emerging as a powerful lens through which to refine precision therapy in leukemia, providing mechanistic insights into how specific alterations in hematopoietic stem and progenitor cells, immune populations, and systemic factors influence both disease evolution and treatment response [121].

At the molecular level, measures such as DNA methylation-based epigenetic clocks, leukocyte telomere length, patterns of cfDNA fragmentation, mitochondrial DNA damage, and plasma proteostasis indicators—including neurofilament light chain and phosphorylated tau provide a window into genomic instability and the biological pace of aging, each of which may shape therapeutic sensitivity [122,123,124,125]. Cellular markers add another dimension: elevated SASP cytokines (IL-6, MCP-1, GDF15), accumulation of p16^+^ immune populations, increased mitochondrial ROS and impaired mitophagy, as well as clonal expansions driven by DNMT3A or TET2 mutations, mechanistically alter HSC differentiation, immune surveillance, and the tumor microenvironment, collectively influencing leukemogenesis and responses to chemotherapy, targeted agents, and immunotherapies [126,127,128]. At the systems level, hallmarks of immunosenescence, chronic low-grade inflammation, defective autophagy, metabolic perturbations (including shifts in IGF-1, FGF21, amino-acid and lipid networks), and microbiome dysbiosis converge to reshape organismal homeostasis, modulating treatment toxicity and resistance through defined molecular and cellular pathways [129].

Collectively, integrative multi-omics approaches—combining genomic, epigenomic, inflammatory, and metabolic data—enable mechanistic dissection of aging-related vulnerabilities, guiding individualized interventions. Such frameworks allow clinicians to tailor treatment based not simply on chronological age, but on biological age and functional reserve, providing mechanistic rationale for forecasting treatment tolerance, relapse risk, and responsiveness to interventions ranging from hypomethylating agents and BCL-2 inhibitors to emerging cellular therapies 2).

## 5. Precision-Medicine Perspectives for Leukemia in the Context of Aging

Population aging has heightened the clinical complexity and biological burden of leukemia. In older adults, unique molecular profiles, immunosenescence, frailty, and comorbidities reduce treatment tolerance and worsen outcomes. Conventional uniform therapies are often inadequate, highlighting the need for approaches tailored to the biology and heterogeneity of this growing patient population.

### 5.1. Harnessing Aging Biology as Therapeutic Targets

Aging imposes distinct vulnerabilities on HSCs and their microenvironment, presenting targets for intervention. Notably, senescent cells accumulate in the bone marrow, releasing a SASP that disrupts HSC function, may promote pre-leukemic clone expansion, and creates a pro-tumorigenic niche.

Preclinical studies demonstrate that pharmacologic clearance of senescent cells using senolytic agents—such as dasatinib combined with quercetin or navitoclax (ABT-263)—can restore hematopoietic function in aged models and mitigate leukemogenic stress in vulnerable marrow compartments [130,131,132,133]. These agents primarily act by targeting anti-apoptotic pathways in senescent cells, thereby enabling their selective elimination and reducing SASP-driven inflammation. For example, dasatinib plus quercetin reduces senescent cell burden in adipose tissue and attenuates systemic inflammation, leading to improved metabolic function in aged mice. Extending these findings, long-term intermittent administration in nonhuman primates lowers inflammatory markers and improves tissue homeostasis, supporting the translational feasibility of senolytic strategies.

Navitoclax (ABT-263), a BCL-2 family inhibitor, has been shown to selectively eliminate senescent cells and rejuvenate aged hematopoietic stem cells. This effect restores self-renewal capacity and enhances hematopoietic reconstitution. Consistently, senolytic clearance also improves the proliferative and differentiation potential of human mesenchymal stem cells, suggesting broader applicability across stem cell compartments.

Recent advances have further expanded this field. Next-generation senolytics, including refined BCL-2 inhibitors and peptide-based approaches, aim to improve selectivity by targeting anti-apoptotic networks more precisely. In parallel, immune-mediated strategies are emerging. These include engineered immune effectors such as CAR-T cells targeting senescence-associated antigens, which enable selective clearance of senescent cells while potentially rejuvenating the bone marrow niche [134].

Overall, senolytic therapies show strong potential to alleviate age-associated hematopoietic dysfunction and inflammation. Further evaluation in elderly acute myeloid leukemia (AML) and CHIP-associated conditions is warranted and may support the development of precision therapies targeting aging biology.

Beyond senescent cell clearance, metabolic dysregulation represents a promising therapeutic avenue. Aged HSCs exhibit impaired NAD^+^-dependent checkpoints, including CD38-mediated NAD^+^ depletion, which contributes to mitochondrial dysfunction and diminished self-renewal. Recent studies show that modulating NAD^+^ metabolism—using CD38 inhibitors, NAD^+^ precursors (e.g., nicotinamide riboside), or mitochondrial stress modulators enhances HSC self-renewal and differentiation, thereby improving resilience to stress and cytotoxic insults [135,136,137]. However, the safety, optimal dosing, and long-term efficacy of these interventions in humans remain largely untested, and potential off-target effects or toxicities have yet to be fully characterized. Early-phase clinical trials are ongoing for some NAD^+^-modulating agents, but their translation from preclinical models to elderly patients with hematologic disorders is still in the exploratory stage. Therefore, while promising, these strategies should be interpreted cautiously until robust clinical evidence is available. Emerging evidence further highlights the broader metabolic rewiring of aged HSCs, including increased oxidative stress, altered mitochondrial dynamics, and dysregulated nutrient-sensing pathways. In particular, aberrant activation of the mTOR signaling pathway has been implicated in HSC aging, and pharmacologic inhibition with agents such as rapamycin has been shown to restore stem cell function and reduce clonal expansion in preclinical models. In parallel, activation of AMPK signaling and improvement of mitochondrial quality control may further support metabolic homeostasis and stem cell maintenance [138]. In addition, metabolic interventions targeting redox balance and lipid metabolism are gaining attention. Elevated reactive oxygen species (ROS) levels in aged HSCs contribute to DNA damage and functional decline, while alterations in fatty acid oxidation and lipid handling can influence stem cell fate decisions. Modulating these pathways may therefore provide additional therapeutic leverage to restore hematopoietic integrity [86].

Epigenetic dysregulation is a hallmark of both CHIP and age-related hematologic disorders, with frequent mutations in DNA methylation and chromatin-remodeling regulators. Therapeutic strategies targeting epigenetic states—including histone deacetylase (HDAC) inhibitors, DNA methyltransferase modulators, and agents that influence chromatin accessibility—have shown efficacy in preclinical models by restraining aberrant gene expression programs and reducing clonal advantage. Furthermore, the aged bone marrow niche itself contributes significantly to hematopoietic decline through altered stromal signaling, accumulation of DNA damage, and loss of supportive factors such as Netrin-1. Notably, supplementation of Netrin-1 in aged mice restores vascular integrity and rejuvenates HSC function, highlighting niche restoration as a viable therapeutic axis. Mechanistically, Netrin-1 acts through its receptor Neogenin-1 (NEO1), which is expressed on HSCs. This signaling helps maintain HSC dormancy and quiescence, thereby linking niche-derived cues to stem cell fate regulation. This mechanistic detail is essential to understand the role of therapies targeting the niche, as interventions that modulate the Netrin 1/Neogenin 1 axis may provide a targeted strategy to restore aged HSC function [27,139]. Additional strategies aimed at modulating niche cytokines, extracellular matrix components, and vascular support are under investigation, with the goal of reconstituting a supportive microenvironment that limits malignant progression while preserving normal hematopoiesis. Despite promising preclinical results, clinical translation in elderly AML or CHIP patients is limited by scarce trial data, potential toxicity with chemotherapy, and unclear patient selection criteria requiring integrated molecular, functional, and geriatric assessment (Table 3).

Targeting the multifaceted effects of aging on HSCs—including senescence, metabolic dysfunction, epigenetic alterations, and niche deterioration—through senolytics, metabolic modulators, and niche-directed interventions holds promise for precision therapies aimed at restoring hematopoietic function and improving outcomes in elderly patients with hematologic malignancies.

### 5.2. Multi-Omics and Computational Frameworks for Aged Hematopoiesis

Building on insights into senescence, metabolism, and niche dysfunction, multi-omics profiling combined with computational modeling offers a roadmap for precision interventions in aged AML and MDS.

Integrative high-resolution profiling is key to deciphering the cellular heterogeneity of aged hematopoiesis and identifying high-risk pre-leukemic clones [91,140,141]. Single-cell multi-omics has revealed vulnerability nodes in aged HSCs and early leukemic clones invisible to bulk analyses. Epigenetic mapping highlights age-associated chromatin changes driving clonal expansion and therapy resistance, while metabolomics points to mitochondrial and NAD^+^ pathway alterations as actionable targets [142].

Large longitudinal cohorts now integrate genomic data with detailed clinical phenotyping, including common comorbidities in older adults such as hypertension, Parkinson’s, Alzheimer’s, and diabetes [143,144]. This enables dynamic risk stratification for progression from clonal hematopoiesis to overt leukemia. Coupled with machine learning and AI-driven models, these cohorts support reconstruction of clonal trajectories, identification of early predictive signatures, and prioritization of preemptive interventions [145]. Recent studies demonstrate that AI-based risk scoring, leveraging combined omics and clinical data, improves early detection of high-risk CHIP and predicts responses to low-intensity therapies [146].

Cross-scale computational frameworks integrating mutational profiles, single-cell dynamics, niche signals, and clinical covariates are increasingly used to generate “virtual patients.” These models enable simulation of therapeutic responses, optimization of dosing for elderly populations, and rational design of combination therapies. Future strategies may include: (i) establishing community-based cohorts with continuous monitoring of age-related risk factors, and (ii) AI-driven risk stratification that combines clinical, molecular, and geriatric data to predict toxicity, response, and survival, guiding the selection of intensive, low-intensity, or palliative regimens. Together, these approaches offer a more precise and adaptive framework for risk assessment and treatment of leukemia in older adults.

### 5.3. Age-Informed Therapeutic Stratification

Care for older patients should extend beyond chronological age to include physical fitness, comorbidities, and molecular characteristics. Pre-treatment geriatric assessments (GA) in older adults with AML are practical and informative, revealing vulnerabilities such as functional limitations, cognitive status, nutritional deficits, and chronic conditions that influence treatment tolerance and prognosis. GA allows categorization of patients as fit, intermediate-fit, or frail, guiding treatment intensity and supportive care.

At the same time, molecular profiling of AML—including gene panels for common mutations, chromosomal analysis, and epigenetic markers—offers essential information for risk stratification and treatment planning [147,148]. By combining GA results with molecular data, clinicians can tailor treatment strategies: standard or high-intensity therapy for fit patients, reduced-intensity regimens for intermediate-fit patients, and palliative or maintenance-focused care for frail patients. Supportive measures such as infection prevention, nutritional support, and physical rehabilitation should be provided alongside therapy as needed [147]. Monitoring patients closely throughout treatment is key. Regular checks of blood counts, minimal residual disease, organ function, and functional or nutritional status allow clinicians to adjust therapy as required, maintaining a balance between effectiveness and tolerability [149]. Decision-support tools, including risk scores and models that consider GA, molecular findings, and clinical factors, can help guide treatment choices. These tools provide additional perspective on likely responses and potential toxicities, supporting more informed and individualized decision-making [98].

Integrating geriatric assessment, molecular profiling, tailored therapies, ongoing monitoring, and decision-support establishes a framework for age-informed care. This approach aligns treatment with both leukemia biology and the patient’s overall health, aiming to maximize benefit while preserving quality of life in older AML patients.

### 5.4. Interdisciplinary Translational Research and Trials

Building on the previously discussed mechanisms and multi-omics discoveries, these insights need to be validated and translated into actionable strategies through interdisciplinary platforms.

Translating precision medicine into tangible benefits for older adults requires interdisciplinary platforms integrating hematology, gerontology, immunology, systems biology, and bioinformatics. Multi-omics studies combined with clinical and functional data have identified aging-informed biomarkers that predict disease progression, therapy tolerance, and complication risk [75,150]. Importantly, integrating Comprehensive Geriatric Assessment (CGA) with molecular and clonal profiling provides a more accurate framework for risk stratification, as the heterogeneity of aging trajectories limits the utility of chronological age alone in guiding intensive treatment or transplantation decisions. Clinical trials should intentionally include older and frail patients, apply geriatric-appropriate endpoints, and test interventions tailored to aging biology. For CHIP carriers, proactive strategies—serial mutation profiling, monitoring inflammation and metabolism, and low-intensity pharmacologic or lifestyle interventions—may prevent progression to overt leukemia [151,152]. Older patients are often underrepresented in early-phase trials, limiting generalizability. Therapy-induced senescence (TIS), while halting malignant proliferation, may promote SASP-driven expansion of residual clones, increasing relapse risk. Dosing, scheduling, and combination therapies must consider comorbidities and polypharmacy. Clear guidelines for patient selection and monitoring are needed to ensure safety and optimize efficacy [153].

Furthermore, it is critical to recognize the role of TIS as a double-edged sword in older patients. While TIS can act as a potent growth inhibitor, halting malignant cell proliferation, it may also create a SASP that fosters residual pre-leukemic or malignant clones, potentially contributing to relapse. Effective “one-two punch” strategies depend not only on inducing senescence through chemotherapy but also on subsequent interventions to clear senescent cells or mitigate SASP effects, thereby maximizing therapeutic efficacy while minimizing adverse consequences [154].

Recent advances in artificial intelligence (AI) and large language models (LLMs) further enhance this framework by integrating multi-omics, clinical, and geriatric data to identify high-risk pre-leukemic clones and estimate the probability of progression from CHIP to acute leukemia with increasing precision. In parallel, emerging “virtual patient” or “digital twin” approaches enable the integration of disease biology, pathophysiology, and pharmacologic responses into computational models, allowing in silico evaluation of drug efficacy and toxicity. Such strategies may reduce exposure of elderly patients to early-phase trial toxicity and accelerate clinical trial design by optimizing cohort selection and reducing control group requirements [155].

Future priorities include (i) validation of aging-informed biomarkers predictive of progression, toxicity, and infection risk; (ii) inclusion of older adults in mechanism-driven trials with geriatric and patient-centered endpoints; and (iii) clinical translation of interventions targeting senescence, metabolic dysregulation, epigenetic alterations, and immunosenescence. Integrating these strategies supports precision, biologically rational, and patient-centered care for older adults with or at risk for leukemia.

## 6. Concluding Remarks and Perspectives

The rapid growth of the aging population has placed hematopoietic aging and its malignant consequences at the forefront of biomedical research. The hematopoietic system, crucial for both blood production and immune defense, undergoes significant functional decline with age. This deterioration undermines organismal homeostasis and creates a permissive environment for leukemia development [156]. Increasing evidence links age-associated changes in HSCs, the bone marrow niche, and immune surveillance to the initiation, progression, and therapy resistance of leukemia in older adults [152].

At the cellular level, aged HSCs expand in number but lose functional quality, displaying reduced self-renewal, diminished long-term repopulating capacity, and a myeloid differentiation bias [75]. Mechanisms underlying HSC aging include cumulative DNA damage, telomere attrition, epigenetic drift, mitochondrial dysfunction, and dysregulated signaling pathways such as mTOR and JAK/STAT, decoupling stem cell quantity from effective function [157]. Concurrently, the bone marrow microenvironment undergoes structural and inflammatory remodeling. Metabolomic and lipidomic analyses of human HSC reveal that choline levels are high in young cells but decline with age and further in acute leukemia; supplementation in vitro restores stemness, highlighting metabolic vulnerabilities [85]. Additionally, the enzyme ELOVL2, essential for very long chain fatty acid synthesis, decreases in aged HSC and immune progenitors, disrupting membrane lipid composition, impairing B-cell development, and accelerating immune aging [158]. These findings link lipid metabolism, chronic low-grade inflammation, and clonal expansion in aging hematopoiesis.

**Figure 5:**
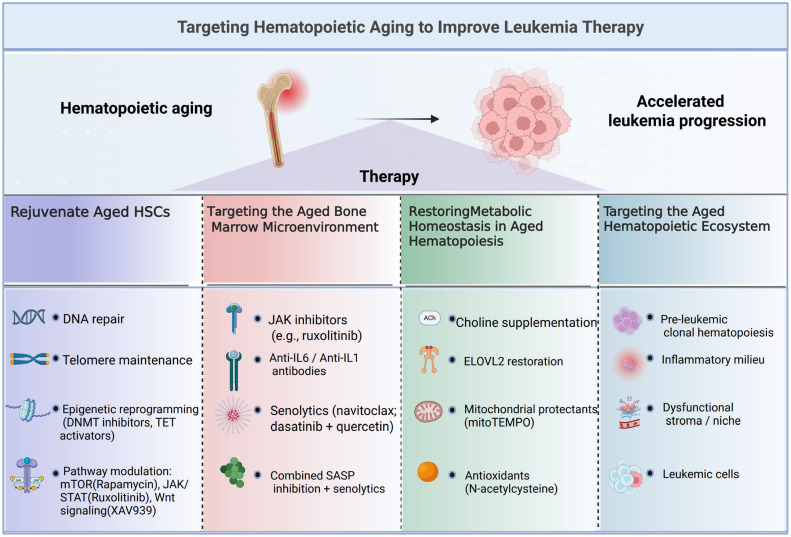
Strategies to Target Hematopoietic Aging for Leukemia Therapy. Overview of approaches to rejuvenate aged hematopoietic stem cells (HSCs) and their niche to slow leukemia. Four strategies: correct HSC defects via DNA repair, telomere stabilization, epigenetic reprogramming, and signaling modulation; rejuvenate the bone marrow niche with JAK inhibitors, anti-inflammatory agents, and senolytics; restore metabolic balance using choline, ELOVL2, mitochondrial protectants, and antioxidants; target the aged hematopoietic ecosystem by addressing pre-leukemic clones, inflammation, niche dysfunction, and leukemic cells. (Created in BioRender. Zhihui Li. (2026) https://app.biorender.com/illustrations/canvas-beta/6916895ab2e34a7feb3a9fbd).

Therapeutically, older leukemia patients face unique challenges. While immunomodulatory antibodies and ICIs are increasingly used, outcomes in older adults are mixed. Some patients benefit, but overall efficacy is often lower than in younger cohorts, due to immunosenescence, comorbidities, and altered pharmacodynamics. Older AML/MDS patients also experience higher rates of serious infections and immune-related adverse events, highlighting the importance of functional and geriatric assessment when considering these therapies. Therapeutic strategies to counter hematopoietic aging and improve leukemia outcomes cluster into four domains. Rejuvenating aged HSCs targets intrinsic defects through enhanced DNA repair, telomere support, epigenetic resetting, and pathway modulation (mTOR, JAK/STAT, Wnt) [93]. Reconditioning the aged marrow microenvironment uses JAK inhibitors, anti-inflammatory antibodies, and senolytics to reduce inflammatory signaling and eliminate senescent cells [159]. Restoring metabolic homeostasis leverages choline supplementation, ELOVL2 recovery, mitochondrial protectants, and antioxidants to correct metabolic and oxidative stress-driven dysfunction [158,160]. Targeting the aging hematopoietic ecosystem addresses higher-level changes-clonal expansion, chronic inflammation, stromal impairment, and leukemic–niche interactions. Together, these approaches underscore that repairing the aging hematopoietic system is central to advancing leukemia therapy (Figure 5). In summary, aging should be recognized as an active and modifiable driver of leukemogenesis, influencing disease initiation, progression, and treatment response across multiple biological scales.

Overall, hematopoietic aging is an active, driving force in leukemogenesis, not a passive backdrop. The future of leukemia therapy for the elderly lies in targeting the aged ecosystem—the pre-malignant clone, the inflammatory milieu, and the dysfunctional niche—in addition to the malignant cells themselves. This requires a concerted interdisciplinary effort across hematology, gerontology, immunology, and computational biology. By reframing aging not just as a risk factor but as a modifiable biological process, we can develop transformative strategies to prevent, delay, and more effectively treat leukemia in our aging population.

## Figures and Tables

**Table 1:** Aging-Associated Biomarkers.

Level & Category	Biological Process	Biomarker	Definition
Molecular Level	Epigenetic Clocks	DNA Methylation Age (HorvathClock, PhenoAge, GrimAge) Epigenetic Age Acceleration (EAA)	DNA methylation-based aging estimators Residual of DNAm age vs. chronological age
	Telomere Dynamics	Leukocyte Telomere Length (LTL) TRF1, POT1 expression	Average telomere length in leukocyte telomere-binding proteins
	Genomic Instability	Cell-free DNA (cfDNA) concentration & fragmentation mtDNA copy number γ-H2AX foci	Circulating DNA integrity Mitochondrial genome abundance Marker of DNA double-strand breaks
	Proteostasis Loss	Plasma Proteome (SomaScan/Olink) NfL (Neurofilament Light Chain)	High-throughput protein profiling Neurofilament light chain (neurodegeneration marker)
Cellular Level	Cellular Senescence	SASP factors (IL-6, MCP-1, PAI-1, GDF15) p16	Senescence-associated secretory phenotype Cyclin-dependent kinase inhibitor
	Mitochondrial Function	ROS production, NAD/NADH ratio Mitophagy flux (PINK1/Parkin)	Reactive oxygen species Cellular redox state Mitochondrial quality control
	Stem Cell Function	CHIP mutations (DNMT3A, TET2), Satellite cell function	Clonal hematopoiesis Muscle regeneration capacity
	Impaired Autophagy	LC3-II, p62 accumulation Beclin-1/ATG deficiency	Autophagic flux markers Autophagy-related genes
Systems Level	Immunosenescence	CD4+/CD8+ ratio, Naïve T T-cell ratio, (IL-6, TNF-α, CRP)	T-cell balance Immune aging marker Inflammatory cytokines
	Chronic inflammation	Macrophages, T cells, B cellsCRP	Prolonged activation of immune cells Systemic inflammation marker
	Metabolome & Nutrient Sensing	IGF-1 HOMA-IR GDF15, FGF21	Growth factor Insulin resistance index Stress/metabolic hormones
	Microbiome	α-diversity, *A. muciniphila*, *E. coli*	Microbial diversity index Beneficial commensal Opportunistic bacteria

**Table 2:** Aging-Associated Mechanisms Contributing to Leukemia Development.

Mechanism Category	Key Features/Changes	Molecular/Cellular Contributors	Impact on Leukemogenesis
Aging-Induced Myeloid Bias	Skewed HSC differentiation toward myeloid lineage; decreased lymphoid output; erythroid output variable	Mutations: ASXL1, RUNX1, SF3B1, SRSF2, CBL; senescent stromal cells; chronic inflammation; oxidative stress	Expands pool of myeloid progenitors prone to malignant transformation; weakens immune surveillance; pre-leukemic clone advantage
DNA Damage & Epigenetic Reprogramming	Accumulation of DNA damage, telomere attrition, replication stress; epigenetic drift (DNA methylation, histone modification, chromatin changes)	Mutations: DNMT3A, TET2, ASXL1; RNA m^6^A modifications; metabolic alterations	Creates permissive environment for clonal expansion; enhances oncogenic potential; promotes leukemic transformation
Metabolic Reprogramming in HSCs/LSCs	Age-associated shift from glycolysis to OXPHOS; increased mitochondrial activity and ROS; LSCs exploit FAO and mitochondrial metabolism	Key enzymes: CPT1A; Tet2 deficiency; altered redox homeostasis and ATP production	Supports LSC survival, self-renewal, clonal dominance, and chemo-resistance; identifies metabolic vulnerabilities for therapy
Bone Marrow Niche	Chronic low-grade inflammation (“inflammaging”); structural/vascular changes; altered stromal metabolism	Elevated cytokines: IL-6, TNF-α, IL-1β; adipocyte expansion; reduced osteogenesis; dysfunctional immune cells (macrophages, T/NK cells); increased regulatory T cells & MDSCs	Impairs normal HSC function; supports LSC survival and expansion; promotes therapeutic resistance
Dependent Clinical Features	Older patients: higher mutational burden, complex karyotypes, therapy resistance; sex differences influence outcome	TP53 mutations, secondary AML (from MDS or prior marrow injury); sex-specific mutations	Alters disease trajectory, prognosis, and therapy response

**Table 3:** Summary of Emerging Therapeutic Strategies Targeting Aging-Associated Hematopoietic Dysfunction.

Therapeutic Strategy	Mechanism/Target	Representative Agents/Approaches	Key Preclinical/Clinical Findings
Senolytic Therapies	Clearance of senescent cells; reduce SASP-driven inflammation	Dasatinib + Quercetin; Navitoclax (ABT-263); next-generation BCL-2 inhibitors; peptide-based senolytics	Restores hematopoietic function, mitigates leukemogenic stress, improves MSC proliferation/differentiation; reduces systemic inflammation
Immunotherapy-based Senolytics	Immune-mediated clearance of senescent stroma	CAR-T cells targeting senescence-associated antigens	Preclinical feasibility demonstrated in aged mice and nonhuman primates
Metabolic Modulators	Restores NAD^+^ levels, mitochondrial function, and metabolic checkpoints in aged HSCs	CD38 inhibitors; NAD^+^ precursors (e.g., nicotinamide riboside); mitochondrial modulators; rapamycin (mTOR inhibitor); AMPK activators; metformin and other metabolic modulators	Enhances HSC self-renewal and differentiation; improves stress resilience; restores mitochondrial quality; reduces ROS; modulates fatty acid oxidation; early clinical exploration in elderly hematologic malignancies
Epigenetic Therapies	Rebalances aberrant gene expression in mutant HSCs and progenitors	(HDAC inhibitors, DNA methyltransferase modulators, chromatin regulators)	Approved for AML/MDS in some contexts; exploration in CHIP models ongoing
Niche-directed Interventions	Restores supportive bone marrow niche; improves vascular integrity and HSC function	Netrin-1 supplementation; modulation of niche ECM, cytokines, vascular factors	Rejuvenates aged HSCs; restores vascular and niche integrity; preserves normal hematopoiesis; limits malignant progression

## Data Availability

The original contributions presented in this study are included in the article. Further inquiries can be directed to the corresponding author.
